# An experimental test of host’s life history traits modulation in response to cuckoo parasitism risk

**DOI:** 10.1371/journal.pone.0179206

**Published:** 2017-06-28

**Authors:** Mónica Expósito-Granados, Deseada Parejo, Juan Gabriel Martínez, Alfredo Sánchez-Tójar, Marta Precioso, Mercedes Molina-Morales, Jesús M. Avilés

**Affiliations:** 1 Department of Functional and Evolutionary Ecology, EEZA-CSIC, Almería, Spain; 2 Department of Zoology, University of Extremadura, Badajoz, Spain; 3 Department of Zoology, University of Granada, Granada, Spain; 4 Evolutionary Biology Group, Max Planck Institute for Ornithology, Seewiesen, Germany; Hungarian Academy of Sciences, HUNGARY

## Abstract

Hosts can counteract parasites through defences based on resistance and/or tolerance. The mechanistic basis of tolerance, which involve defensive mechanisms minimizing parasite damage after a successful parasitic attack, remains poorly explored in the study of cuckoo-host interactions. Here, we experimentally explore the possibility that the risk of great spotted cuckoo *Clamator glandarius* parasitism may induce tolerance defences in magpie *Pica pica* hosts through plasticity in life-history traits. We predict that magpies exposed to auditory cues indicating high parasitism risk will more likely exhibit resistance and/or modify their life-history traits to minimize parasitism costs (i.e. tolerance) compared to magpies under low parasitism risk. We found that manipulating the perceived parasitism risk did not affect host resistance (i.e. rejection of parasitic eggs) nor host life-history traits. Unexpectedly, host’s egg volume increased over the season in nests exposed to auditory cues of control non-harmful hoopoes *Upupa epops*. Our results do not provide support for inducible defences (either based on resistance or tolerance) in response to risk of parasitism in magpie hosts. Even so, we encourage studying plastic expression of breeding strategies in response to risk of cuckoo parasitism to achieve a better understanding of the mechanistic basis of tolerance defences.

## Introduction

Parasitism is a strong selective agent shaping host evolution [1]. Plant and animal hosts can minimize the negative effects of parasites through defences based on resistance and/or tolerance [2–6]. Resistance involves physiological or behavioural defensive mechanisms that minimize the likelihood of being parasitized whereas tolerance involves defensive mechanisms reducing the negative effect of the parasite after a successful attack [6]. Contrary to resistance, tolerance diminishes the impact of parasitism without necessarily causing a negative effect on the parasite [6,7]. Although plant biologists have long discriminated between resistance and tolerance when studying plant defences [6,8], studies of animal enemy-victim interactions on tolerance defences are still scarce [6,8,9], particularly those investigating the mechanisms behind tolerance (but see [10]).

In birds, interspecific brood parasitism is a reproductive strategy in which the parasite (e.g. cuckoo) lays eggs in the nest of another species, the host, which carries out the parental duties from incubation to offspring feeding [11,12]. Brood parasitism often imposes large fitness costs to hosts, for example, due to egg removal or breakage during egg laying by the parasitic female, eviction of host eggs and/or nestlings from the nest by the parasite chick, and starvation of host’s offspring when the parasite chick monopolizes parental feeds [11,13]. As a consequence, natural selection has favoured the evolution of certain behaviours preventing effective parasitism by cuckoos (i.e. resistance mechanisms) ranging from mobbing cuckoos before laying (e.g. [14,15,16]), to discrimination and rejection of cuckoo eggs (e.g. [17–20]), or nestlings (e.g. [21,22]).

Beyond defences based on resistance, avian brood parasites may have selected for host tolerance as well [6]. Defences based on resistance can be costly because hosts may incur in recognition and rejection errors [23] or because parasites may destroy host eggs as a punishment to host resistance [24,25]. This scenario is likely to promote the evolution of tolerance defenses, which are by definition less costly [9]. Theoretical reviews have largely emphasized the need of simultaneously studying resistance and tolerance when assessing host defences against cuckoo parasites [6,9]. So far only the study by Soler et al. [26] has explicitly tested the adaptive value of tolerance while accounting for resistance in the Great spotted cuckoo *Clamator glandarius*-Eurasian magpie *Pica pica* host system. Operational tolerance (i.e. the slope of the regression between the number of cuckoo eggs in a clutch and the number of host produced fledglings) was larger in highly parasitized magpie populations, suggesting that tolerance may have evolved as an adaptive response to great spotted cuckoo parasitism [26].

However, empirical studies about the mechanisms of tolerance are lacking, possibly due to the more subtle and less detectable manifestation of tolerance mechanisms at the population level. Several indirect sources of evidence suggest that some hosts may reduce the costs of parasitism (i.e. show some degree of tolerance) by, for example, increasing clutch size [27,28] or laying less eggs but in several nesting attempts [29–31]. Although the evolutionary causes of these different strategies remain poorly studied, the use of one strategy or another appears to be contingent on whether brood parasites are raised together with host nestlings or alone [9]. Furthermore, these studies were not designed to detect tolerance, and given their correlative nature they do not allow discriminating if changes in host life-history traits in response to parasitism reflected a micro-evolutionary process or were due to phenotypic plasticity.

Plastic responses regarding resource allocation to environmental stress are among the most commonly studied tolerance mechanisms in plants (reviewed in [32,33]), but their role is still poorly understood in animal enemy-victim interactions. Variable risk of predation can induce plasticity in life-history traits in birds [34–37]. Also, cuckoo hosts can flexibly change resistance defences (e.g. nest defence [15,38], egg rejection [17,39–41] and nestling rejection [42]) as a response to the risk of brood parasitism. However, it remains unknown whether variable risk of cuckoo parasitism may also induce plasticity in host life-history traits, and whether that plasticity may help somehow to minimize the costs of cuckoo parasitism.

In this study, we investigated host defences in a cuckoo-host system, the Eurasian magpie—Great spotted cuckoo, hereafter cuckoo, at the intra-population level. Theoretical work has suggested that this would be a suitable system for the evolution of defensive mechanisms based on tolerance [6,9] given that in this system rejection costs are particularly high due to the retaliatory behaviour of the brood parasite. Here we experimentally modified the risk of cuckoo parasitism during laying and incubation of magpies and measured their response in terms of egg rejection (i.e. as a proxy of resistance) and plasticity of life-history traits (i.e. number of eggs and their volume) that may subsequently influence host fitness (i.e. tolerance).

Previous theoretical studies had suggested the existence of a trade-off between resistance and tolerance mechanisms, so that if one of the two defences evolves, then the other would be less likely to do it [6]. However, empirical evidence suggests that this would not apply to our study system as resistance does not covary with tolerance across magpie populations [26]. We predict that individual magpie hosts under high risk of suffering brood parasitism will be more likely to exhibit resistance (i.e. more prone to reject cuckoo eggs) and/or tolerance (i.e. more prone to modify their life-history traits to minimize the costs of raising cuckoos) compared to individuals under low risk of parasitism. In birds, females may modify their breeding investment to buffer variable environmental conditions affecting their offspring prospects. This can be achieved through changes in number and volume of eggs [43], and therefore it is worth exploring whether a modification in perception of risk of parasitism may induce different host breeding investment (i.e. clutch size and egg volume).

## Methods

### Study area and system

The study was conducted in La Calahorra (37° 10´ N, 3° 03´ W, Hoya de Guadix, Southern Spain). This is a patchy area of approximately 12 km^2^ where groves of almond trees *Prunus dulcis*, in which magpies preferentially build their nests, are very common [44,45]. Magpies are territorial, sedentary, and socially monogamous long-lived passerines [46]. In our population, the great spotted cuckoo is a non-mimetic egg and specialist parasite of the magpie, although can sporadically parasite other corvids as the Hooded crow *Corvus corone* [47]. Great spotted cuckoo females usually destroy magpie eggs while laying and multiple parasitism is frequent [48]. Once hatched, the parasite chicks do not evict the magpie nestlings, but due to their shorter incubation length and faster development of the cuckoo, magpie nestlings rarely fledge from parasitized nests [49]. The natural parasitism rejection in magpie hosts is about 5% [24], lower than that for experimental models of cuckoo eggs (see [50]). The percentage of parasitized nests in our population (i.e. parasitism rate) greatly varies between years (range 15.9–65.6%, [51]).

### Experimental manipulation

We conducted a manipulation of cuckoo parasitism risk during the laying and incubation periods of magpies in the year 2014 (mid-March to April). The breeding season of this year lasted from the first egg laid on mid-March to end of June when the last nestlings fledged. At the beginning of the breeding season magpie nests were located by careful inspection of all trees in the area, and GPS positioned. In total, 75 nests were found, but 21 nests were discarded from the experiment because the laying activity had already finished when they were found. The remaining 54 nests were found before clutch completion (mean ± SE clutch size when found was 3.13 ± 0.23 eggs (range: 1–7), final clutch size of magpies in our population: Parasitized nests (mean ± SE) = 6.56 ± 1.11 (range: 4–9); Non-parasitized nests (mean ± SE) = 6.85 ± 0.76 (range: 5–9), [45]). Each nest was randomly assigned to one of the following treatments: i) "increased parasitism risk": perceived risk of cuckoo parasitism was increased by broadcasting cuckoo calls in the surroundings of the nest (n = 23 nests), ii) "no parasitism risk": calls of Hoopoes *Upupa epops* were played as a control for the effect of the playback (n = 14 nests); and iii) "control": we visited the surroundings of the nest as often as for treatments i) and ii), but did not play any call (n = 17 nests).

Portable amplified speakers (MOLGAR 3” 20W 4 ohm) connected to digital audio players (takeMS MP3 Player “Deseo”) were used to broadcast calls. The calls consisted of three different tracks of great spotted cuckoo calls and four hoopoe calls from different individuals extracted from Llimosa et al. [52] and three 1-min silence tracks, respectively, that were randomly selected and played continuously using the random function in the audio players during the experiment. This produces unique assortments of calls by their randomized presentation and combination with silence tracks for each nest and treatment, thus minimizing the risk of pseudoreplication [53,54]. Treatments were applied three times during two hours every third day from the day each nest was found. Some nests were found at the very beginning of laying whereas others did not, and, hence, every nest might have received either 1 or 2 playback sessions during laying. However, the number of eggs laid at the time we started our experiment did not vary among treatments (see below Variables and Experimental Randomization). The minimum distance between two magpie nests in this study year was 350 m, thus minimizing the possibility that magpies others than the tested perceived the stimulus.

Previous experimental studies have shown that birds have the potential to perceive threats based on acoustic cues emitted by predators (e.g. [53,55]), and even avian brood parasites (*Chalcites* cuckoo species [56] and Brown-headed cowbird (*Molothrus ater*) [57]), which justifies the only use of vocal cues for manipulating parasitism risk. Furthermore, male cuckoos perch and call close to host nests to attract magpie attention; meanwhile female cuckoos make a silent approach to the nest to lay [58]. Therefore, continuous broadcasting of cuckoo calls close to a nest is likely to be perceived as a real parasitism challenge for magpies. We chose the hoopoe as a “no parasitism risk” control because hoopoe *a priori* poses no threat to magpies and lives in sympatry with them.

We opted to manipulate risk of parasitism once laying was started in the knowledge that in birds, females have the potential to modify their investment on eggs (i.e. hormone composition and egg size) in response to a sudden change in environmental conditions during laying [59–62].

### Estimation of host resistance

On the first visit to each nest, we introduced a plaster model egg that resembled a cuckoo egg in appearance, size and mass (see details in [44]). Previous studies have shown that rejection of model eggs provides a reliable estimate of host defences based on resistance [26,63]. We determined level of resistance as a categorical variable with two levels: rejecter, if the model egg disappeared from the nest, and acceptor, if the model egg was still in the nest after 6 days. Although rejecter magpies reject experimentally added eggs within the first 72 hours [64], we chose here a longer response period to avoid modifying magpie perception of risk of parasitism if we had removed accepted eggs at day three, as this might have affected life-history traits of those pairs that would have not yet complete the clutch.

### Variables and experimental randomization

For all nests we recorded number of laid eggs (before and after the experiment), and egg volume (before and after the experiment). 10 nests were discarded because either have unusual small clutch sizes (3 or less eggs) which suggested partial predation (n = 6 nests), or were totally predated (n = 4 nests). We measured the length and width of each egg with a digital calliper; egg volume was calculated using the formula Volume = Length * Width^2^ * 0.515 [65].

To test whether the experiment was fully randomized with respect to magpie pair quality we checked for differences before the experiment in magpie laying date (a proxy of individual quality in magpies [66]) and average magpie egg volume already laid between our three treatments using one-way ANOVAs. Neither laying date (*F*_2,51_ = 0.33, *P* = 0.72) nor the average egg volume recorded before the experiment (*F*_2,45_ = 0.81, *P* = 0.45) differed among treatments, suggesting that our experiment was properly randomized regarding magpie quality. Additionally, we tested for differences in number of laid eggs the day of the experiment (i.e. before any effect of broadcasting) using a generalized linear model with a Poisson distribution. Number of laid eggs before the experiment did not differ between the treatments (*F*_2,51_ = 0.13, *P* = 0.88), suggesting that treatments were evenly established regarding magpie laying sequence.

### Statistical analyses

To study the effect of risk of parasitism on magpie resistance, we ran a generalized linear model (GENMOD procedure in SAS) in which probability of egg rejection was entered as a binary dependent variable (rejection *vs*. acceptance; link function: logit) and treatment as fixed factor with three levels (increased parasitism risk, no parasitism risk, control). Laying date (1 = 1^st^ March) was fitted as a covariate to control for possible differences in magpie quality. Additionally, we fitted the interaction of laying date and treatment. Given that previous work has shown that egg rejection behaviour increased with age in female magpies [50], we conducted a second model including a subset of 23 females of known age. In this model, age was entered as a fixed factor.

To check for the effect of the parasitism risk on magpie life-history traits, we tested for differences among treatments in number of eggs laid after the experiment (Poisson distribution, link = log) and in mean egg volume after the experiment (normal distribution, link = identity) using a generalized linear model (GENMOD procedure in SAS) and a general linear model (GLM procedure in SAS), respectively. Again, treatment, laying date, and the interaction between the two were fitted as predictors. Graphical inspection of residuals plots indicated that the error distribution of the data was modelled correctly and did not depart from Poisson and normal model assumptions, respectively. To improve the interpretability of regression coefficients laying date and egg volume were mean-centred.

Analyses were performed in SAS, version 9. 4.

### Ethical statement

Consejería de Medio Ambiente y Ordenación del Territorio (Junta de Andalucía) authorized the fieldwork of the present study (projects CGL2011-27561/BOS and CGL2014-56769-P; licence code: P06-RNM-01862). Spanish law does not require ethical approval for this specific study from an International Animal Care and Use Committee (IACUC). Experimental manipulation of perceived parasitism risk did not affect the natural rate of nest abandonment of the species suggesting that our experimental procedure has a negligible negative effect on magpies.

## Results

### Risk of parasitism and host resistance

Magpies rejected 20 out of 54 (37.03%) model eggs; however, the risk of cuckoo parasitism did not influence egg rejection behaviour once we control for laying date (rejection rates were 34.78%, 35.71% and 41.17% for the increased risk of cuckoo parasitism, no-risk of cuckoo parasitism and control treatments, respectively(Treatment effect: *χ*^2^_2_ = 0.14, *P* = 0.93; laying date effect: χ^2^_1_ = 0.19, *P* = 0.67; Treatment*laying date: χ^2^_2_ = 0.58, *P* = 0.75). A second model only with the subset of females of known age confirmed this pattern (Treatment effect: *χ*^2^_2_ = 0.36, *P* = 0.84; laying date effect: χ^2^_1_ = 0.65, *P* = 0.42; Treatment*laying date: χ^2^_2_ = 1.57, *P* = 0.46; Age effect: χ^2^_1_ = 0.53, *P* = 0.47).

### Risk of parasitism and host life-history traits

**Fig 1:**
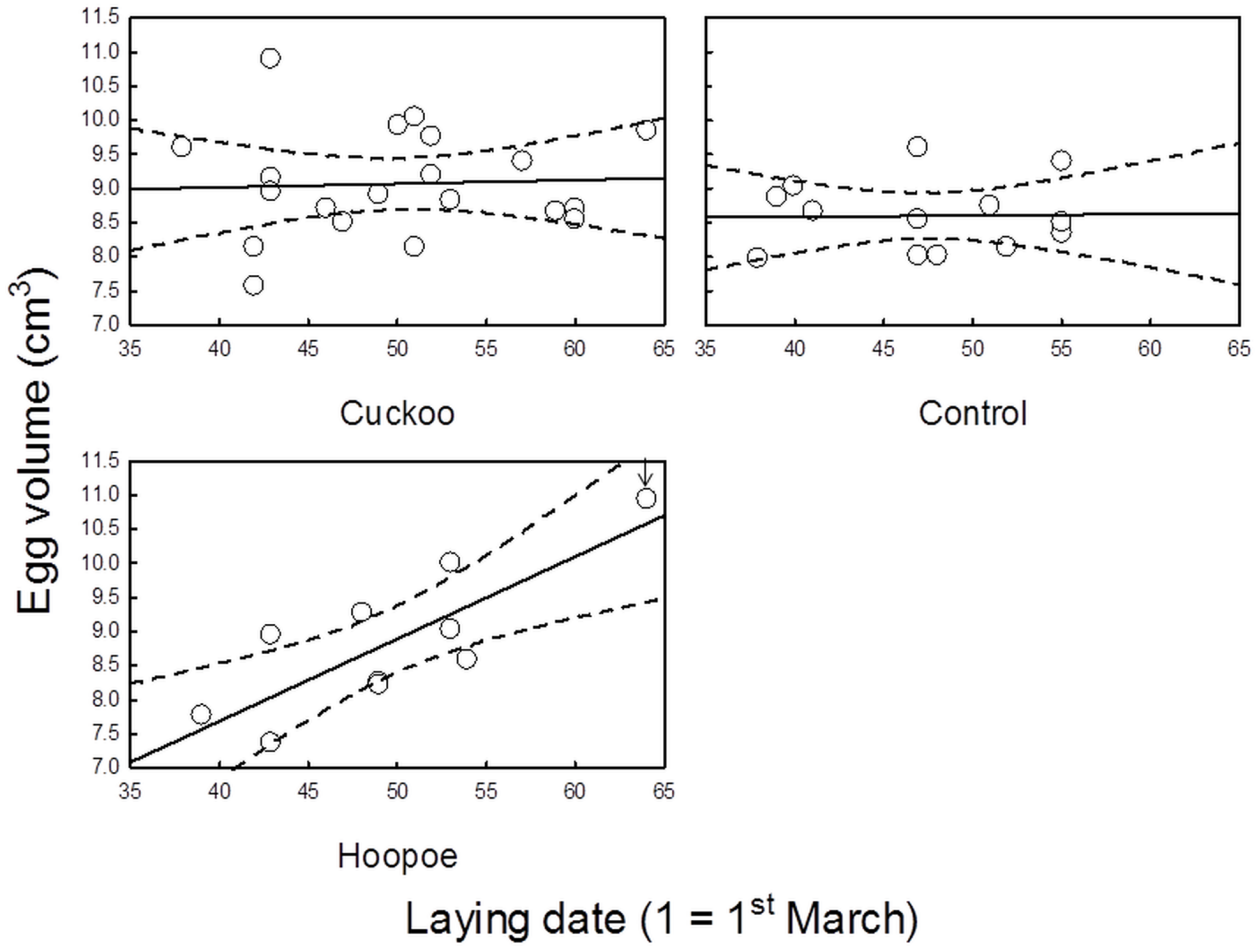
Seasonal variation in volume of magpie eggs laid after the experiment in relation to risk of parasitism at the nest. Dashed lines represent the 95% confidence limits for the regression line (solid line). Egg volume and laying date were centred for the analysis but not in the figure to allow a biological interpretation of measurements. The arrow indicates an *a priori* outlier.

Magpies did not modify their investment in number of eggs after the experiment, and this pattern did not vary over the season (Table 1). However, there was a significant effect of treatment on average magpie egg volume, which changed over the season (Table 2). Egg volume after the experiment increased over the season in nests exposed to the hoopoe treatment, whereas it did not vary in nests exposed to the cuckoo treatment or in control nests (Fig 1). The interactive effect of treatment and laying date on egg volume remained robust when we excluded the control group of nests in which host life-history traits could not be measured after 25^th^ April due to logistic problems (see Fig 1; Laying date*treatment: *F*_1,26_ = 6.68, Coefficient (low CL, high CL) = 0.005 (0.001, 0.009), *P* = 0.02; Laying date: *F*_1,26_ = 8.57, Coefficient (low CL, high CL) = 0.0003 (-0.002, 0.003), *P* = 0.01; Treatment: *F*_1,26_ = 6.91, Coefficient (low CL, high CL) = -0.59 (-1.06, -0.13), *P* = 0.01). However, this pattern vanished when we excluded the late extreme value in host egg volume in the hoopoe treatment (Fig 1), which nonetheless cannot be considered a statistically significant outlier (Grubb’s test: G = 2.62, P > 0.05, n = 43).

**Table 1:** Variation in number of magpie eggs in relation to risk of cuckoo parasitism at the nest.

Number of eggs (n = 44 nests)							
Term		Coefficient	Lower CL	Higher CL	*F*	DF	*p*
Intercept		0.85	0.48	1.21	4.71	40	<0.0001
Laying date		-0.02	-0.05	0.001	3.69	1,40	0.06
Treatment	Hoopoe	0.5	0.03	0.98	2.46	2,40	0.09
	Cuckoo	0.39	-0.05	0.83			
	Control*						
Laying date*treatment	Hoopoe	-0.06	-0.14	0.02	1.12	2,38	0.34
	Cuckoo	-0.03	-0.1	0.04			
	Control*						

Results of generalized linear model of number of magpie eggs laid after the experiment in relation to risk of cuckoo parasitism.

CL, 95% confidence level.

* Reference level in the treatment contrast parametrization of the model matrix.

**Table 2:** Variation in magpie egg volume in relation to risk of cuckoo parasitism at the nest.

Egg volume (n = 43 nests)							
Term		Coefficient	Lower CL	Higher CL	*F*	DF	*p*
Intercept		-0.28	-0.79	0.12	-1.42	37	0.16
**Laying date**		0.002	-0.06	0.07	**6.06**	**1,37**	**0.02**
Treatment	Hoopoe	0.17	-0.44	0.79	1.68	2,37	0.2
	Cuckoo	0.46	-0.06	0.99			
	Control*						
Laying date*treatment	Hoopoe	0.12	0.02	0.21	**4.65**	**2,37**	**0.02**
	Cuckoo	0.003	-0.08	0.08			
	Control*						

Results of general linear model of volume of magpie eggs laid after the experiment in relation to risk of cuckoo parasitism. Significant terms are highlighted in bold.

CL, 95% confidence level.

* Reference level in the treatment contrast parametrization of the model matrix.

## Discussion

Recent theoretical work has emphasized the importance of integrating the study of resistance and tolerance defences to better understand the evolution of animal enemy-victim interactions, including those between avian brood parasites and their hosts [6,8,9]. Despite this, empirical studies focusing on tolerance defences in cuckoo-host systems are surprisingly scarce and, so far only confined to investigate operational tolerance across different host populations [26]. Furthermore, the mechanistic basis of tolerance still remains unstudied in the framework of cuckoo-host interactions.

Here we explore the possibility that tolerance may be expressed at the population level through plasticity in host life-history traits. However, we found that manipulating the perceived risk of cuckoo parasitism, did not affect the rejection behaviour of parasitic eggs (a reliable indicator of host resistance against cuckoo parasites) nor host life-history traits which are candidate to reflect tolerance. However, our experimental set-up revealed plastic expression of magpie host life-history traits in response to experimental treatments at the nest. Magpies did not modify the number of eggs laid but egg volume.

Magpies did not change their resistance against cuckoos in response to the risk of parasitism at their nests. Previous studies had reported the existence of plastic resistance (rejection and mobbing behaviour) in hosts of the European cuckoo *Cuculus canorus* in response to risk of parasitism [17,39–41,67–69]. Plastic resistance was explained as an adaptive response to the high cost of rejecting and mobbing in populations where risk of parasitism greatly varied in space and time [15]. Our findings, however, agree with Soler et al. [70], who found that placing a live cuckoo close to a magpie nest did not modify the rate of ejection of cuckoo model eggs. Experimental results in the magpie-cuckoo system documented that cuckoos destroy magpie eggs after realizing that magpies have rejected their eggs (Mafia hypothesis, [24]). Therefore, one likely explanation for the lack of effect of risk of parasitism on egg rejection is that the costs of rejecting when there is constant presence of cuckoos in the surroundings are greater than those of accepting the parasitic egg. However, this would predict a stronger response in hoopoe and control nests, which is not the case. Another possibility is that most of our tested magpies could have been young individuals, which, in contrast to adult females, may still have not learnt to reject cuckoo eggs or perceive parasitism risk and respond accordingly [50]. This possibility, however, is unlikely because most females of known age (78.26% of N = 23 females) during the experimental year were adults (older than two-years old). Within this sample, female mean age was 4.04 years old (range: 1–8). Moreover, our results confirmed the absence of a treatment effect on rejection once we control for female age. Alternatively, it could be argued that our playback experiment may have failed to increase perceived risk of parasitism but, instead, worked as an additional non-important parasitic stimulus added to the introduced model egg. This could have been the case if magpies were already aware of having been (artificially) parasitized, which would just make cuckoo calls an extra threat. On the other hand, it is possible that the stimulus needed to provoke a response is the association of call and cuckoo presence together but not cuckoo call (as in this study) or cuckoo presence (as in [70]) separately. However, it is unlikely that magpies disregarded call information as they did react to hoopoe calls (see below).

Magpies did not modify their life-history traits (number and egg volume) in response to the risk of cuckoo parasitism. Unexpectedly, they modified the volume of their eggs in response to hoopoe calls, which is intriguing given previous work showing that hoopoes were considered as a non-harmful threat by magpies [71]. Egg volume increased over the season in nests exposed to hoopoes but not in nests exposed to cuckoos or in control nests. One first explanation for the absence of an effect of cuckoo calls could be that magpies might have already perceived their nests as being parasitized because we introduced a model cuckoo egg, and hence, they might disregard cuckoo calls. Another possibility could be that broadcasting was performed too late in the laying of the host with no time to induce a physiological response. However, these two possibilities seem very improbable because magpies responded to hoopoe calls.

Regarding the unexpected effect of hoopoe calls on egg volume, several explanations are possible. First, although hoopoes do not predate on magpie nests it is possible that hoopoe calls emitted close to the nest would have been perceived as a predation threat by magpies because hoopoes calls may draw the attention of predators to the nest [72]. In this scenario high quality breeders, which reproduce early in the season, reduced their investment in current reproduction by laying smaller eggs when perceiving a potential risk, as they could still save some energy for a replacement clutch [73]. Alternatively, hoopoe calls may have attracted territorial hoopoe males in the surroundings of magpie nests. Indeed, we detected in some instances the presence of active singing males in the neighbourhood of nests where hoopoe songs were broadcasted (Pers. Obs.). The unusual and active presence of hoopoe males (in addition to calls) close to their nests, could have led to changes in their life-history traits due to the additional source of stress. Also, it could be the case that late breeding magpies associated hoopoe presence with factors other than risk, as high-quality habitats and/or food abundance. Last, but not least, given the low sample size in the hoopoe treatment, it cannot be discarded that this pattern emerged randomly and corresponded with a false positive result or a methodological artefact due to an unexpected effect of the used control stimulus [74].

## Conclusions

We have theoretically introduced and experimentally tested for the first time the possibility that hosts may tolerate cuckoo parasitism through plastic expression of reproductive traits. The reasoning for this novel hypothesis is based on overwhelming evidence documenting plastic responses in resource allocation in response to environmental stress in plants (reviewed in [32,33]), and a large body of empirical work showing that birds may plastically modify their breeding strategies in relation to perceived risk of predation at their nests [37,72,73]. However, we did not find that magpies modified their breeding strategy in response to risk of parasitism. Although our results do not support the tolerance hypothesis, we emphasized the need of studying plastic expression of breeding strategies in response to different sources of environmental stress in hosts of avian brood parasites, and, their fitness consequences for the parasites. In this sense, it seems advisable to study the same reproductive couple throughout a season or the same individuals in different seasons (i.e. using longitudinal studies) to achieve a better understanding on how hosts may tolerate cuckoo parasitism.

## Supporting information

S1 File Data of experimental test of host’s life history traits modulation in response to cuckoo parasitism risk. (XLSX)
